# Four climate change scenarios for *Gypsophila bermejoi* G. López (*Caryophyllaceae*) to address whether bioclimatic and soil suitability will overlap in the future

**DOI:** 10.1371/journal.pone.0218160

**Published:** 2019-06-13

**Authors:** Miguel de Luis, Julio Álvarez-Jiménez, Juan Manuel Martínez Labarga, Carmen Bartolomé

**Affiliations:** 1 Departamento de Ciencias de la Vida. Facultad de Biología, C. Ambientales y Químicas Universidad de Alcaláo, Alcalá de Henares (Madrid), Spain; 2 Departamento de Sistemas y Recursos Naturales, E.T.S.I. Montes, Forestal y del Medio Natural, Universidad Politécnica de Madrid, Madrid, Spain; University of Michigan, UNITED STATES

## Abstract

Climate change has altered the global distribution of many species. Accordingly, we have assessed here the potential shift in the distribution of *Gypsophila bermejoi* G. López under distinct scenarios of future climate change, this being a species endemic to the Iberian Peninsula. For strict gypsophiles, climatic changes affecting their potential area of distribution could be critical if the new range is not overlapped with suitable soils. Thus, the narrow bioclimatic niche and the endemic nature of this plant could make this species particularly vulnerable to climate change. We used the Maximum Entropy (MaxEnt) method to study the potential distribution of this taxon under four different scenarios of climate change, pin-pointing relevant changes in the potential distribution of this plant and enabling possible future areas of refuge to be assessed. Such scenarios are defined according to four Representative Concentration Pathways (RCPs) [, which represent different trends in the concentration of atmospheric carbon dioxide. As a result, we predict notable changes in the potential distribution of *G*. *bermejoi*, and the overlap between soil and bioclimatic suitability would be affected. We also used a Principal Component Analysis (PCA) to model the bioclimatic niche of this species, comparing it with that of its parental taxa. The evolution of bioclimatic suitability was assessed at the current locations of *G*. *bermejoi* and as this plant is a strict gypsophile, we generated suitability maps for sites with gypsum soils. Ultimately, this study identifies relevant changes in the potential distribution of *G*. *bermejoi* under specific climatic scenarios, observing remarkable differences in the outcomes of the different climate change scenarios. Interestingly, in some scenarios the bioclimatic suitability of *G*. *bermejoi* will be enhanced at many locations and even in the worst scenario some possible refuge areas were identified. *G*. *bermejoi* behaves more like a hardy survivor than as early victim.

## 1- Introduction

The average global temperature has increased greatly over the last 100 years and a higher rate of warming has been projected in the future [1]. Shifts in temperature have affected the habitats of a wide range of species [2] and accordingly, climate change has already altered the distribution of species in several regions around the world [3]. Shifts in the range of some plant species have been highly significant, averaging 6.1 km per decade towards the poles, and a significant mean advancement of spring events by 2.3 days per decade has also been recorded [4].

While these changes may be associated with a higher risk of extinction and a loss of biodiversity, biological responses to such changes would be expected. For instance, plants may display changes in species population genetics, altered species richness on a local scale or shifts in ecotone boundaries **[5,6,7]**. The rate of such change is an important concern, and it will be crucial to determine the fate of the species affected and their ecosystems. Most responses to such climate change involve phenotypic plasticity or adaptations in the species’ phenology. Such plasticity may increase environmental tolerance and enhance the ecological spread of a species **[8–10]**. Moreover, the advancement of spring events and a significant lengthening of the vegetative growing season **[11,12]** may also produce different phenological responses between interacting species. For example, the asynchrony between insect and plant systems might increase, usually with negative consequences **[13]**.

As a result, it is crucial to assess the magnitude of possible extinction events, as has been attempted through a series of different approaches. The risk of extinction can be assessed by projecting species distributions in the face of future climatic conditions. Indeed, some mid-range predictions of climate-warming for 2050 suggest that 15–37% of species and taxa in the regions sampled will be ‘committed to extinction’ **[14,15]**. This loss of biodiversity will not be equal in all regions of the world and the Iberian Peninsula, along with the rest of the Southern Mediterranean Basin, may be very vulnerable due to synergies between climate change and other anthropic factors. In fact, it is important to consider land use when performing such studies in order to obtain more accurate projections **[16]**.

Mediterranean plants may be particularly susceptible to global warming because they are currently near their optimum temperature and thus a further increase in temperature would almost certainly affect their growth and survival **[17,18]**. Significantly, different projections predict a lower frequency of rainfall in the Mediterranean basin and extreme drought during the summers. Such threats may be even greater for small-range species **[19]**, and the relationship between range size and extinction risk has been highlighted in a number of studies. For range-restricted species, stochastic events (local catastrophic events, droughts or disease outbreaks, etc.) or enhanced land transformation will affect larger proportions of the total populations, increasing the risk of extinction. For instance, polar and mountain-top species have undergone severe range contractions **[20]**, and they are among the first groups in which entire species have become extinct or could face extinction in the short term due to anthropic climate change **[13]**. Nevertheless, the situation of endemic plant communities on special soils has received little attention to date. For example, ultramafic areas harbor large numbers of endemic species, ecotypes and rare species, and hence, they are considered high priority areas for biodiversity conservation **[21]**. Such consideration may also be given to other plant communities found on special soils, like gypsum, limestone, dolomite and shale **[22]**.

Some strict gypsophile species endemic to the Iberian Peninsula might also be under threat due to the consequences of climate change. Thus, here we set out to assess whether this phenomenon will be likely to drive these species out of their suitable soils, focusing specifically on the case of *Gypsophila bermejoi* G. López. In addition to previous considerations regarding plants in a Mediterranean climate, this plant might be at a higher risk for two reasons: firstly, it is a strict gypsophyte and, as such, the areas in which these plants can prosper are limited; secondly, this species displays a particularly narrow range of bioclimatic conditions, more so than other endemic Iberian *Gypsophila* species. Indeed, this is reflected in a recent detailed study comparing the bioclimatic niches of *G*. *bermejoi* and its parental taxa **[23]**.

Gypsisols have more than 15% gypsum (calcium sulfate dihydrate, CaSO_4_ 2H_2_O **[24–26]**, the solubility of which is relatively low, and hence, the vegetation associated with these areas is more similar to that of calcareous soils than to saline soils **[25]**. Indeed, the osmotic potential of these soils does not increase very much and its ion specific toxicity is extremely low **[27]**. Water availability in gypsum soils must also be considered, as their poor water retention could produce important deficits during the summer months **[25, 28]**. There is abundant literature on the plant life associated with gypsum soils **[29–33]**, the presence of a hard surface soil crust representing an important feature explaining the restrictive nature of these soils for plant establishment **[34]**. Nevertheless, gypsophytes like *G*. *bermejoi* face other problems, such as macronutrient deficiencies, ionic antagonism (Ca^2+^, Mg^2+^) or macronutrient toxicity **[34–35]**.

It is difficult to assess the risk of extinction in the case of plant species adapted to special soils as their survival depends on the existence of suitable soils within an area of climatic suitability, areas that may shift as a result of climate change. Plant communities on special soils like gypsisols or serpentine soils have two distinctive attributes that make difficult to assess their response to climate change. Not only does their spatial isolation make migration more difficult when these species have to adjust their distribution but also, these are plants that already display traits that are highly adapted to stress tolerance **[22]**.

In the present study we aim to test if *G*. *bermejoi* has a narrower climatic niche than its parental taxa, *Gypsophila struthium* L. subsp. *struthium* and *Gypsophila tomentosa* L., making this species more vulnerable to climate changes. We will plot the niche models by means of density plots. In addition, we studied the changes in the bioclimatic suitability area of this species under four different climatic scenarios, each defined according to four Representative Concentration Pathways (RCPs) **[36–38]**. Species Distribution Models **[39]** allow the distribution of species to be predicted under different climatic scenarios. In this way, we could assess if areas of future bioclimatic suitability for this gypsophyte are likely to overlap with suitable soil locations **[19, 22]**. We will use the Maximum Entropy (MaxEnt) method for this purpose. Such information could be crucial to locate potential future refuges for this species, enabling new conservation strategies to prioritize the protection of such areas **[40]**.

## 2- Materials and methods

The potential distribution of *G*. *bermejoi* was first obtained on the basis of the bioclimatic variables and later, we studied the suitability of the habitat at the sites where the soil favors these species. We did this for the current climatic conditions, and with the projections for 2050 and 2070. In order to locate suitable sites, we used data on the presence of *Ononis tridentata* L., a common and widespread species that lives on more or less pure gypsum soils and on gypsiferous loams **[41,42]**.

### 2.1- Study area and species

This study focuses on the gypsum outcrops of the Iberian Peninsula, included in an area close to 623,000 km^2^ that is located between latitudes 36°00’08"N—43°47’38”N and longitudes 9°29’00"O—3°19’00”E. The gypsum steppes in this territory have a patchy distribution, with a total extension of 32,487 km^2^ (6.1% of the Peninsula) **[31]**, and they are concentrated in the eastern half of the Peninsula under the influence of a Mediterranean climate. The gypsum soil habitat is generally structured as plant patches interspersed on an exposed ground matrix, with well-developed biological soil crusts dominated by eukaryotic algae, cyanobacteria, mosses, liverworts, fungi and lichens **[43–44]**. The spatial structure of gypsum outcrops reflects an island-like configuration, providing interesting opportunities to study evolutionary processes, such as plant speciation **[24,45]**.

**Fig 1:**
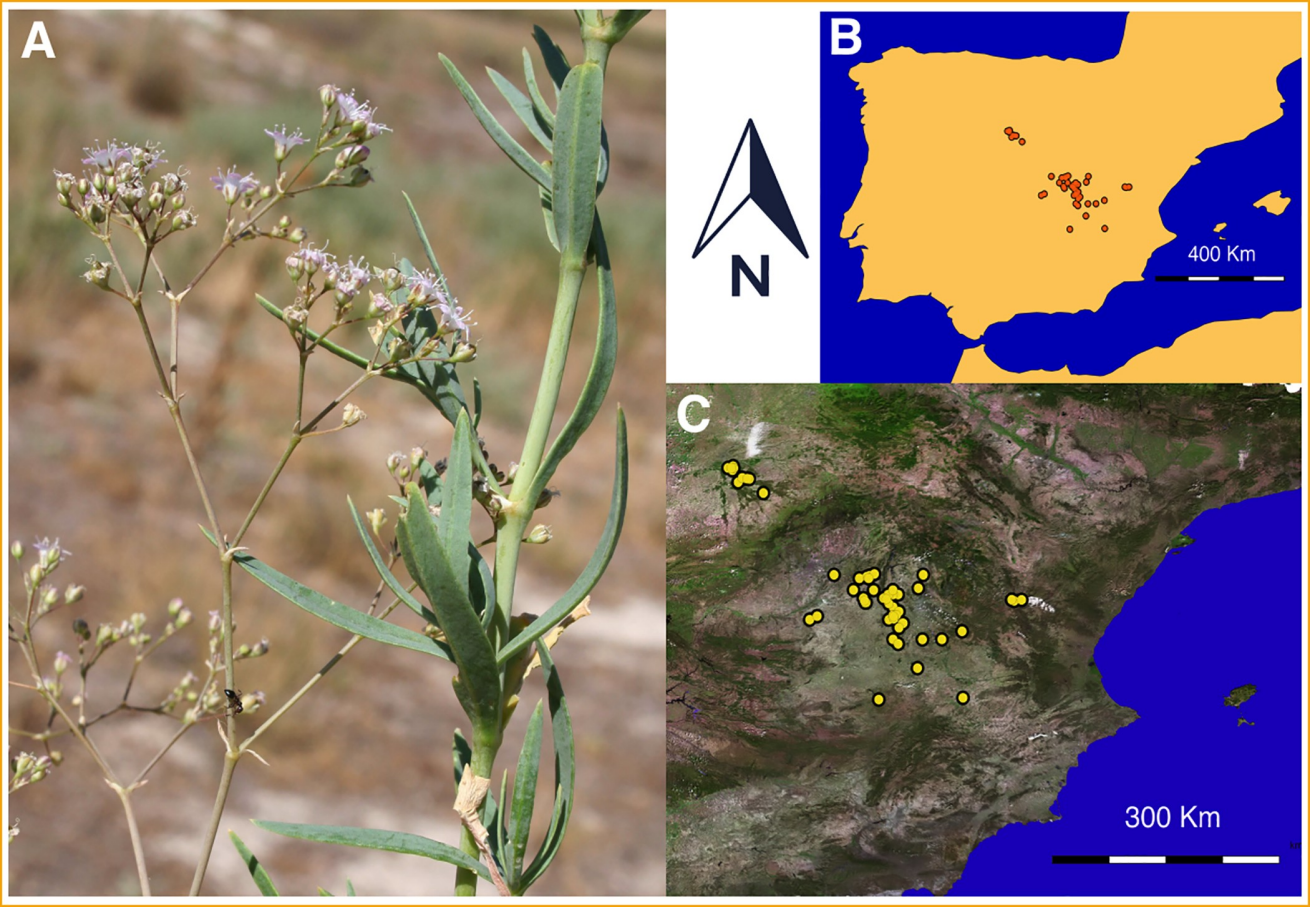
Appearance of *G*. *bermejoi* (A). Maps of the Iberian Peninsula showing where this species can be found (B and C) according to GBIF data.

*Gypsophila bermejoi* (2n = 68) is a hybrid endemic species of the Iberian Peninsula that thrives in areas of gypsum soil. This allopolyploid species is the result of a natural cross between two wild species, *G*. *struthium* subsp. *struthium* (2n = 34) and *G*. *tomentosa* (2n = 34) **[46]**, and its bioclimatic range is narrower than its parents’ **23]**. Each of these perennial plants are endemic to the Iberian Peninsula, inhabiting salt-rich substrates and fundamentally, gypsum soils. *G*. *bermejoi* is a tetraploid, perennial, suffruticose, strict gypsophile with glabrous, flat leaves (the inferior ones 15–50 × 2–8 mm) containing 1 to 3 nerves. The small pale pink flowers are arranged in corymbiform cymes **[46]** (Fig 1), and the ovary has 2–5 carpels and styles, usually containing numerous ovules arranged in a central placental column. The seeds are small (1.1–1.5 mm), black and reniform, and they can be released from the capsules by the action of animals or strong winds **[47]**. In addition, an opportunistic strategy of germination has been observed in both parental taxa, with water availability likely to be the principal limitation to seed germination and plant establishment **[48]**.

### 2.2- Data sources

We used the data on species occurrence from the Global Biodiversity Information Facility (GBIF) database **[49].** These data consist on downloadable csv (comma separated values) files including information concerning the collection process and species' taxonomy, with geographical information (if any) provided as longitude and latitude values or the locality. The information for *O*. *tridentata* (3388 registers) and *G*. *bermejoi* (122 registers) was filtered to remove non georeferenced registers and some obvious mistakes. The bioclimatic variables were downloaded from The WorldClim portal (www.worldclim.org), where data for 19 bioclimatic variables are publically available **[50]**. Maps of future values of these bioclimatic variables are also available on this platform, generated using different climate models. Here, we have used the bioclimatic maps generated by the Community Climate System Model (CCSM4) for 2050 (as the average of 2041–2060) and for 2070 (averaged from 2061–2080). Four separate models of the atmosphere, land, oceans and sea-ice, are unified in this model, generating a simulation of the global climate **[51]**.

The maps for 2050 and 2070 were modelled under four different climate change scenarios or RCPs, scenarios that describe four possible trajectories for CO_2_ emissions and atmospheric concentrations between 2000 and 2100. The different output curves reflect different assumptions for a range of variables, including human population size, energy consumption and land use **[36–38]**. The most benign scenario of anthropogenic climate change is RCP 2.6, in which global CO_2_ emissions peak by 2020 and then fall to values around zero by 2080. In addition, the peak atmospheric concentration is delayed by approximately 30 years after the peak emissions. In the RCP 4.5 scenario, peak emissions occur around 2050, declining rapidly over the next 30 years and then stabilizing. The CO_2_ concentration also increases but it begins to fall after 2070. In RCP 6, the CO_2_ emissions double by 2060 and they then plummet, although they remain much higher than the current levels. In this scenario, the atmospheric concentrations of this gas still increase, although they do so more slowly towards the end of the century. RCP 8.5 is by far the worst scenario, with CO_2_ emissions increasing at a high rate during the first part of the century and while they stabilize by 2100, the concentrations of CO_2_ are over three times those in 2000 **[36–38]**.

### 2.3- Maximum entropy (MaxEnt) model

The potential distribution of *G*. *bermejoi* was modeled under the four climate change scenarios using the Maximum Entropy (MaxEnt) method **[52,53]**. We used the most recent version of the program available (MaxEnt 3.4.1), with a graphical interface (this method is explained in detail in **[54, 55]**). In general terms, this algorithm assesses the environmental suitability probability of a species being present as a function of the environmental variables established. Data regarding the distribution of the species and a series of maps of the relevant environmental variables are used as input, and the program extracts the values of each variable for all the points where the species is present. As such, the distribution obtained is determined by the preferences of the species and this can be extrapolated to the remaining areas of study. These data can also be extrapolated to different territories, or even to different moments in the past or future, although this requires executing climatic models that allow maps to be elaborated with the variables estimated for these specific moments in the past or in the future. The probability distribution that adjusts to the model or the statistics used in MaxEnt satisfy the so-called principle of maximum entropy. As such, it is assumed that the values estimated for the habitat required by the species are obtained through the superposition of the points of presence in the maps of the environmental variables. Therefore, the distribution obtained for the probability of presence does not assume anything else about the distribution a priori from these variables. In other words, this distribution maximizes the value of the Shannon index (a measure of the entropy) given the restriction of the values of known presence **[52–55]**.

The output values can be interpreted as an index of suitability to the habitat for a particular species **[55]**. We dealt with autocorrelation issues by eliminating the redundant pixels on the scale of the bioclimatic variables used. We used 70% of the distribution records to train the models and 30% to test them. This study focused on the effect of climatic oscillations and as such, we only used bioclimatic variables to generate the models. Indeed, the strict gypsophillic nature of these plants mean they are only found on gypsum soils.

When elaborating the models using the MaxEnt method, the correlation between the predictors should be minimized **[55]**. From the set of environmental variables we eliminated those that are strongly correlated using a clustering algorithm. A dendrogram was obtained that grouped the variables through their degree of correlation, based on a Pearson coefficient. Furthermore, we ran the models with all the variables to perform a jack-knife test on the contribution of the variables to the model to select those with the highest contribution from the dendrogram.

Sampling bias is an important issue when using MaxEnt. The datasets of species occurrence could be biased in the geographical space due to unequal sampling effort across the study area **[56]**. Albeit, we consider that the sampling process has proportionately covered the full range of environments in the region due to the limited range in an endemic species as *G*. *bermejoi*. This circumstance, according to Phillips et al., would not cause problems if the MaxEnt model is based on environmental data **[57]**. Spatial autocorrelation is other concern repeatedly reported when using SDM **[58]**. However, some studies comparing its effect with other important factors, like sample size, sampling design and modeling technique, have revealed that spatial autocorrelation has only comparatively small effects **[59]**.

After combining the information obtained through correlation dendrogram and jackknife for variable contribution, and taking into account the abiotic factors determining the vegetation patterns in a semi-arid Mediterranean landscape **[60]**, we selected from the less correlated variables those with the highest contribution, and one related with precipitation values during the dry season **[60]**.(Table 1).

**Table 1:** List of the environmental variables selected to implement the MaxEnt species distribution models for *G*. *bermejoi*.

Variable	Source	Resolution
**Bio 4**—seasonal temperature dispersion	WorldClim	30”
**Bio 6** –minimum temperature in the coldest month	WorldClim	30”
**Bio 13** –rainfall in the wettest month	WorldClim	30”
**Bio 14** –rainfall in the driest month	WorldClim	30”

The models were evaluated using two validation methods: the Area Under the Curve (AUC) and the True Skill Statistics (TSS) **[61,62]**. In choosing the functions “feature”, we included linear, quadratic and product functions. In this way, the response curves obtained were easier to interpret and they were better adjusted to the unimodal response curves predicted by niche ecological theory **[55]**.

### 2.4- Bioclimatic niches and occurrence density curves

We used the approach developed by Broennimann *et al*. (2012) **[63]** to study the bioclimatic niche of *G*. *bermejoi* and its parental taxa. Specifically, we used an ordination method to determine the occurrence density along two environmental axes. In this case, we selected the same bioclimatic variables used in the MaxEnt models.The first two axes of this PCA analysis were used to define the environmental space, which is limited by the minimum and maximum environmental values found across the whole study area. We then divided the environmental space into a grid of 100 x 100 cells, each cell corresponding to a specific combination of environmental values. Subsequently, a kernel smoothing method was used to plot the species density on this gridded environmental space. This approach enables us to use an algorithm implemented in R language **[64]** to plot the species niches in pairs in order to make their comparison easier **[65]**.

We also obtained occurrence density curves for the bioclimatic factors one by one. This is very useful as it makes it possible to assess the tolerance ranges and compare them between two different taxa. The R package ‘Ecospat’ **[66]** was used to carry out all these analyses.

### 2.5- The evolution of suitability values at sites where *G*. *bermejoi* is currently found

The main goal of this study was to evaluate the possible shift in bioclimatic suitable areas out the gypsum soils, due to climate change. In other words, we set out to determine whether bioclimatic suitability and soil availability will overlap in the future. One way to answer this question is to study the evolution of the bioclimatic suitability values at the sites where *G*. *bermejoi* is currently found. We used a Geographical Information System (QuantumGIS **[67]**) to generate maps of the sites where *G*. *bermejoi* is currently found, showing its bioclimatic suitability using a color code. These maps can be displayed as a sequence (for the present climatic conditions, and those in 2050 or 2070), indicating whether the bioclimatic conditions change over time and if they improve or worsen. While the information gained in this way is easy to interpret, it is less precise because the values are grouped in intervals. To overcome this limitation, we also generated a sequence of box-plots that depict the changes in bioclimatic suitability over time.

### 2.6- Study of suitability at gypsum soil sites

The MaxEnt output maps show the changes in bioclimatic suitability within the study area. Nonetheless, it is known that the absence of adequate soil conditions would make the true suitability zero in most of the territory. To assess suitability in the gypsiferous areas, we need to know their locations. Outcrops of this type do not commonly cover large extensions and over the scale of the variables used, most of these outcrops would appear as mere points. Therefore, to trace the areas with gypsum soils we chose to use records of the presence of a gypsum indicator plant, *Ononis tridentata* **[24,68–69]**. This species was used as sole gypsum soils indicator for the several reasons: it is one of the most abundant gypsophyles in the Iberian Peninsula and this species thrives in a wide range of bioclimatic stages and gypsum soils. This species can be found on the Thermomediterranean (TMM), Mesomediterranean (MSM) and Supramediterranean (SPM) stages. It is also present on all types of gypsum soils, including crusts, saccaroid soils and gypsum marls, sometimes with low gypsum content. *O*. *tridentatata* is the gypsophile with the lowest of gypsum content requirements and, as such, it can be present even in some washed soils of the Supramediterranean (SPM) stage. Again, we used QuantumGIS **[67]** to represent the georeferenced presence of *O*. *tridentata* and the pattern of the data obtained coincided perfectly with the published maps of Iberian gypsum soils **[24]**. Subsequently, we obtained the suitability values from the models for those locations at three points in time: now, 2050 and 2070. Again, these values were color coded in the maps and through box-plots.

It is important to stress that high values of bioclimatic suitability at the locations with suitable soils do not necessary mean the presence or colonization by *G*. *bermejoi* at that site. We know that is very probable that past climatic changes have driven the migration of this taxon but we need to know much more about the capacity of this species for dispersal.

## 3- Results

From the MaxEnt models generated for the species studied, the AUC value obtained was 0.965, high enough to validate the models. The TSS value for the same species was 0.810, with values above 0.6 considered to be good and those of 0.2–0.6 to be fair to moderate.

### 3.1- Predicted changes in the potential bioclimatic areas by 2050 and 2070 under the four RCP scenarios

**Fig 2:**
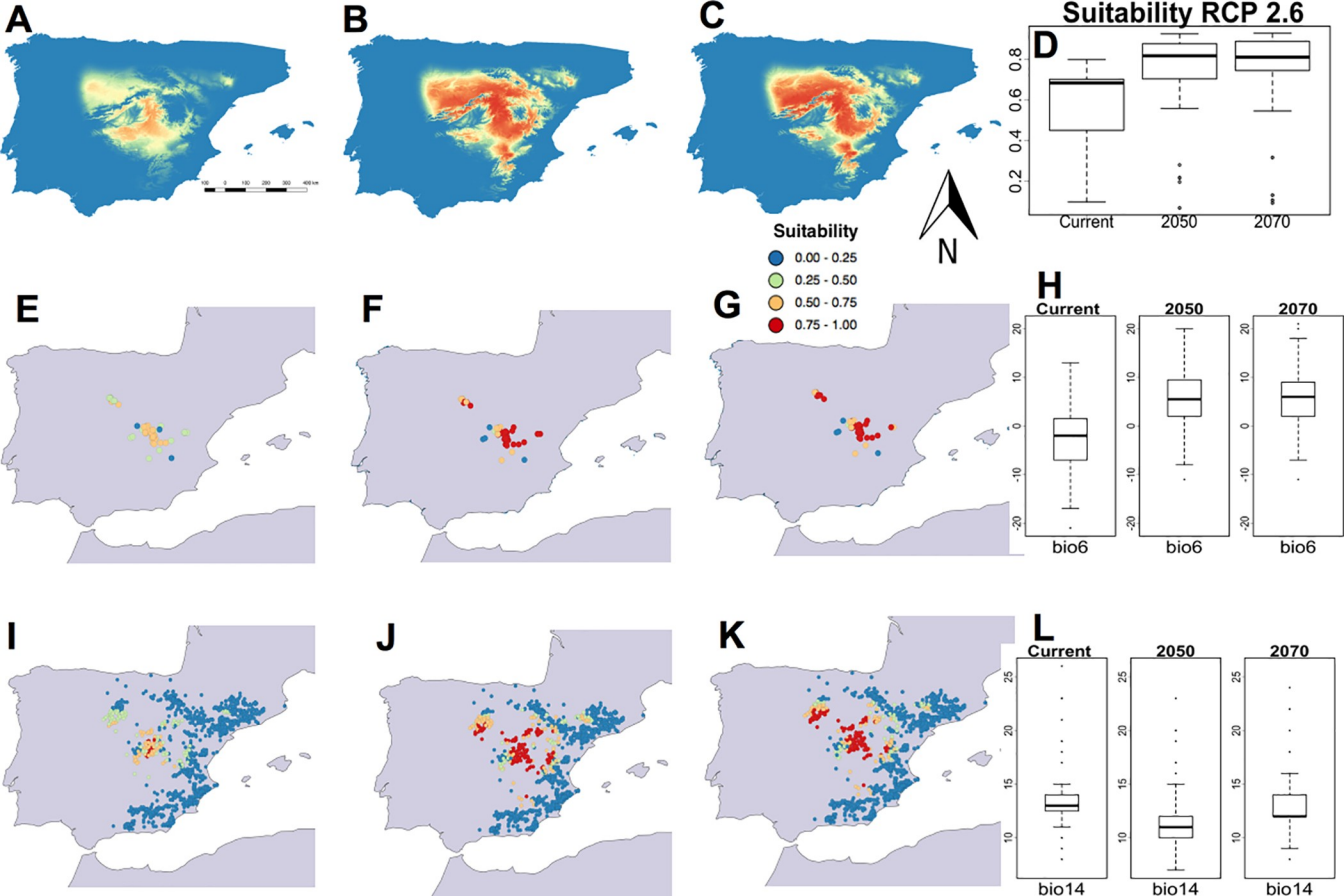
Habitat suitability predicted by the MaxEnt models for *G*. *bermejoi* under the RCP 2.6 scenario. Habitat suitability map for the current climatic conditions (A), those predicted for 2050 (B) and those for 2070 (C). (D) Habitat suitability values in the current climatic conditions (R software standard box-plot), and under those predicted for 2050 and 2070, measured at the sites where this taxon is currently found. In E, F and G, we show the bioclimatic suitability values for the sites where *G*. *bermejoi* is currently found. To aid interpretation of these maps, a color code was used to express the different intervals of habitat suitability. (H) These box-plots show the evolution of the values for the Bio 6 variable (the minimum temperature in the coldest month) measured at the sites where *G*. *bermejoi* is currently found. The bioclimatic suitability was also measured at each location with gypsum soil indicated by the presence of *O*. *tridentata*. These values are shown for the current climatic conditions, and the conditions predicted for 2050 and 2070, in maps I, J and K, respectively. (L) These box-plots are similar to those in (H) but for the values of the Bio 14 variable (precipitation in the driest month), also measured at the locations where *G*. *bermejoi* is currently found.

Under the RCP 2.6 scenario, the potential bioclimatic area for *G*. *bermejoi* was predicted to have expanded by 2050, stabilizing thereafter (Fig 2A to 2C). The bioclimatic suitability values for *G*. *bermejoi* at its current locations seemed to improve noticeably, following a similar trend. In general terms, this scenario enhanced the bioclimatic suitability of this species at its current locations (Fig 2D and 2E to 2G), the median value shifting from its current value of 0.6811 to a higher median of around 0.8171 by 2050, thereafter remaining stable until 2070 (0.8263: Fig 2D and Table 2). We also examined the bioclimatic variables relative to temperature and rainfall (Bio 6 and Bio 14), which usually show an opposite trend (Fig 2H and 2L). The variations observed in Bio 6 could be important as the median moved from values below 0 ºC to positive temperature values. The rainfall during the driest month (Bio 14) decreased, with a median value slightly above 10 mm in 2050 and 2070. We focused on these particular variables because they are ecologically relevant and easy to interpret. Considering all the sites with gypsum soils in the Iberian Peninsula, this scenario could favor strong bioclimatic suitability for this taxon at some locations where it is currently not found (Fig 2J and 2K and Table 2).

**Table 2:** Evolution of the median values for the selected bioclimatic variables under the RCP 2.6 scenario. The evolution of the suitability medians is also shown on the bottom row. All the values are taken at the current locations of *G*. *bermejoi*.

RCP 2.6			
**Median**	**Current**	**2050**	**2070**
**Bio 4**	6612	7112	7071
**Bio 6**	-2.000	5.500	6.000
**Bio 13**	54.00	50.00	50.00
**Bio 14**	13.00	11.00	12.0
**Suitability**	**0.6811**	**0.8171**	**0.8263**

**Fig 3:**
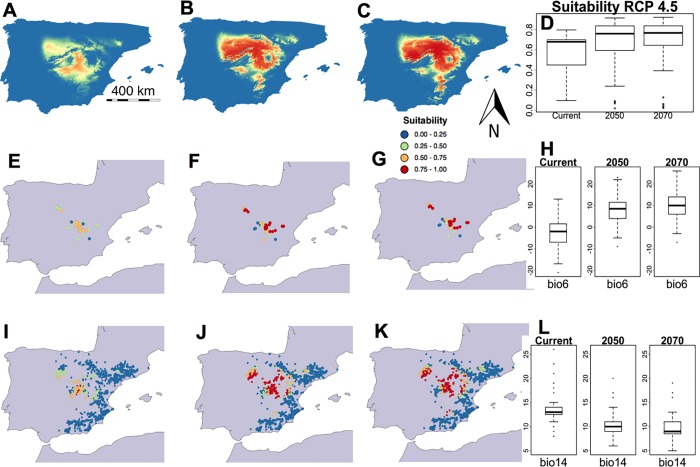
Habitat suitability predicted by the MaxEnt models for *G*. *bermejoi* under the RCP 4.5 scenario. Habitat suitability map for the current climatic conditions (A), and those predicted for 2050 (B) and 2070 (C). (D) Habitat suitability values in the current climatic conditions, and under those predicted for 2050 and 2070 (R software standard box-plot), measured at the sites where this taxon is currently found. In E, F and G, we show the bioclimatic suitability at the sites where *G*. *bermejoi* occurs at present. In these maps, a color code was used to express the different intervals of habitat suitability. (H) These box-plots show how the Bio 6 variable values evolve (minimum temperature in the coldest month) at the sites where *G*. *bermejoi* is currently found. The bioclimatic suitability was also measured at each location with gypsum soil indicated by the presence of *O*. *tridentata*. These values are shown for the current climatic conditions, and under the conditions predicted for 2050 and 2070, in maps I, J and K, respectively. (L) These box-plots are similar to those in (H) for the values of the Bio 14 variable (precipitation in the driest month), also measured at the locations where *G*. *bermejoi* is currently found.

The situation predicted by the RCP 4.5 conditions showed a less intense increase in suitability than under RCP 2.6 (Table 3). While the Bio 6 values were slightly higher, those of Bio 14 were slightly lower for the 2050 and 2070 projections (Table 3). Nevertheless, there seemed to be no significative differences between the trends in bioclimatic suitability (Fig 3D), although they increased at most of the current locations of this taxon (Fig 3E to 3G). The most remarkable difference is that there were more sites where *G*. *bermejoi* is not currently found that will adopt favorable bioclimatic conditions (Fig 3, maps 3I to 3K). Indeed, one difference with respect to RCP 2.6 is that there were more locations with better bioclimatic conditions around the Ebro Valley, a region where this species is not currently found.

**Table 3:** Evolution of the median values for the selected bioclimatic variables under the RCP 4.5 scenario. The evolution of the medians of suitability are also shown in the bottom row. All the values are taken at the current locations of *G*. *bermejoi*.

**RCP 4.5**			
**Median**	**Current**	**2050**	**2070**
**Bio 4**	6612	7231	7365
**Bio 6**	-2.000	8.500	10.00
**Bio 13**	54.00	48.00	45.50
**Bio 14**	13.00	10.00	9.00
**Suitability**	**0.6811**	**0.76141**	**0.7692**

**Fig 4:**
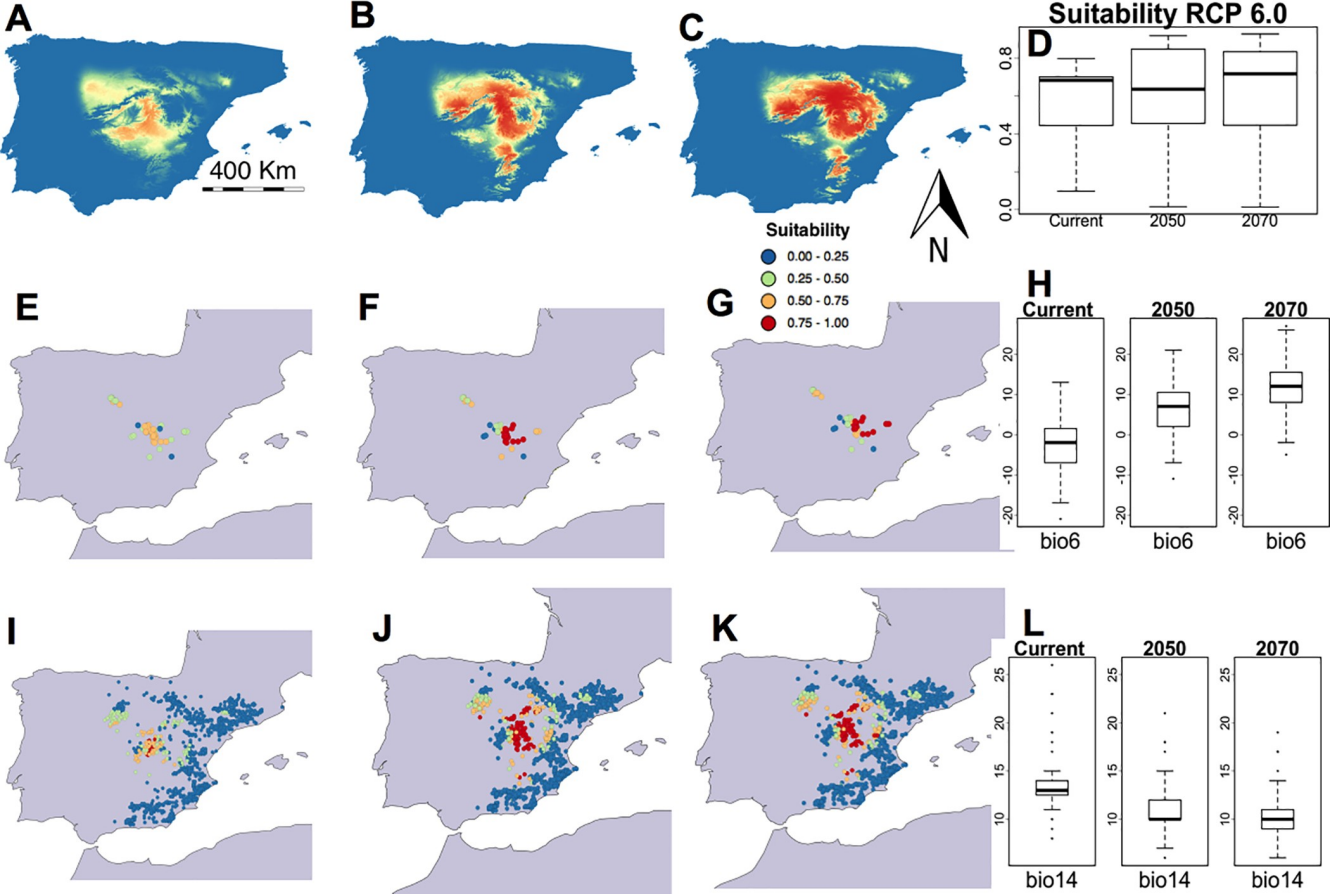
Habitat suitability predicted by the MaxEnt models for *G. bermejoi* under the RCP 6 scenario. Habitat suitability map for the current climatic conditions (A), and for those predicted for 2050 (B) and 2070 (C). (D) Habitat suitability values (R software standard box-plot) in the current climatic conditions, and under those predicted for 2050 and 2070 measured at the sites where the taxon is currently found. In E, F and G, we show the bioclimatic suitability at the sites where *G*. *bermejoi* is currently found. A color code was used to express the different intervals of habitat suitability. (H) These box-plots show the evolution of the values of the Bio 6 variable (the minimum temperature in the coldest month) measured at the sites where *G*. *bermejoi* is currently found. Bioclimatic suitability was also measured at each location, with gypsum soils indicated by the presence of *O*. *tridentata*. These values are shown in maps I, J and K, for the current climatic conditions, and for the conditions predicted for 2050 and 2070, respectively. (L) These box-plots are similar to those in (H) but representing the values of the Bio 14 variable (precipitation in the driest month), also measured at the locations where *G*. *bermejoi* is currently found.

A different situation was evident in the RCP 6.0 scenario, with bioclimatic suitability values increasing at some sites where *G*. *bermejoi* is found, whereas the median suitability values seemed noticeably lower than in the RCP 2.6 and RCP 4.5 scenarios. There was a decrease in the median value from 0.6811 (the current value) to 0.6495 by 2050, increasing thereafter to 0.7287 by 2070 (Table 4). This climatic scenario reflected a difference in the evolution of the Bio 6 and Bio 14 values, Bio 6 values increasing further after 2050 and those of Bio 14 continuing to diminish (Fig 4H and 4L). Under such circumstances, there is no noticeable increase in bioclimatic suitability among the northwestern populations of *G*. *bermejoi* (Fig 4E to 4G), although the maps of the *O*. *tridentata* locations reveal some of the sites where bioclimatic suitability will increase (Fig 4I to 4K).

**Table 4:** Evolution of the medians for the selected bioclimatic variables under the RCP 6.0 scenario. The evolution of the suitability medians is also shown on the bottom row. All the values are taken at the current locations of *G*. *bermejoi*.

RCP 6.0			
**Median**	**Current**	**2050**	**2070**
**Bio 4**	6612	7135	7358
**Bio 6**	-2.000	7.000	12.00
**Bio 13**	54.00	51.00	47.0
**Bio 14**	13.00	10.00	10.00
**Suitability**	**0.6811**	**0.6495**	**0.7287**

**Fig 5:**
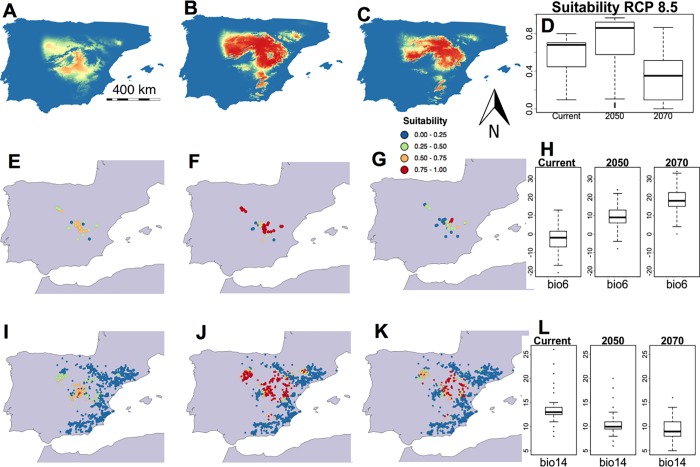
Habitat suitability predicted by the MaxEnt models for *G*. *bermejoi* under the RCP 8.5 scenario. Habitat suitability map for the current climatic conditions (A), and under those predicted for 2050 (B) and 2070 (C). (D) Habitat suitability values (R software standard box-plot) in the current climatic conditions, and under those predicted for 2050 and 2070 measured at the sites where the taxon is currently found. In E, F and G, we show the bioclimatic suitability values taken at the sites where *G*. *bermejoi* is currently found. A color code was used to express the different intervals of habitat suitability. (H) These box-plots show the evolution of the Bio 6 variable (minimum temperature in the coldest month) measured at the sites where *G*. *bermejoi* is currently found. The bioclimatic suitability was also measured at each location for gypsum soils indicated by the presence of *O*. *tridentata*. These values are shown in maps I, J and K, for the current climatic conditions, and under the conditions predicted for 2050 and 2070, respectively. (L) These box-plots are similar to those in (H) but for the Bio 14 variable (precipitation in the driest month), also measured at the locations where *G*. *bermejoi* is currently found.

There was a dramatic contrast between the results obtained under scenario RCP 8.5 and those associated with the other scenarios. By 2050, the predicted bioclimatic suitability area seemed to have expanded and moved northwards. By 2070 this area had again diminished, although it remained at a higher latitude. In this case there was a smaller overlap between the bioclimatic suitability and gypsum soils (Fig 5A to 5C), and consequently, the bioclimatic suitability median at the locations of *G*. *bermejoi* increased until 2050 (0.8600), thereafter dropping abruptly at 2070 (0.3518: Fig 5D to 5F and Table 5). Many locations with very high suitability values (red) are shown in the projection for 2050, a scenario that changed dramatically by 2070, with most of the current locations of *G*. *bermejoi* adopting very low bioclimatic suitability values (blue). In the maps of all the gypsum soil sites, high suitability was evident in many places in 2050 although again, this changed significantly in 2070 when the high bioclimatic suitability values in the northwestern region decreased notably (Fig 5I to 5K).

**Table 5:** Evolution of the medians for the selected bioclimatic variables under the RCP 8.5 scenario. The evolution of median suitability is shown on the bottom row. All the values are taken at the current locations of *G*. *bermejoi*.

RCP 8.5			
**Median**	**Current**	**2050**	**2070**
**Bio 4**	6612	7487	7739
**Bio 6**	-2.000	9.00	18.00
**Bio 13**	54.00	46.00	45.00
**Bio 14**	13.00	10.00	9.00
**Suitability**	**0.6811**	**0.8600**	**0.3518**

### 3.2- Bioclimatic niches and occurrence density curves

**Fig 6:**
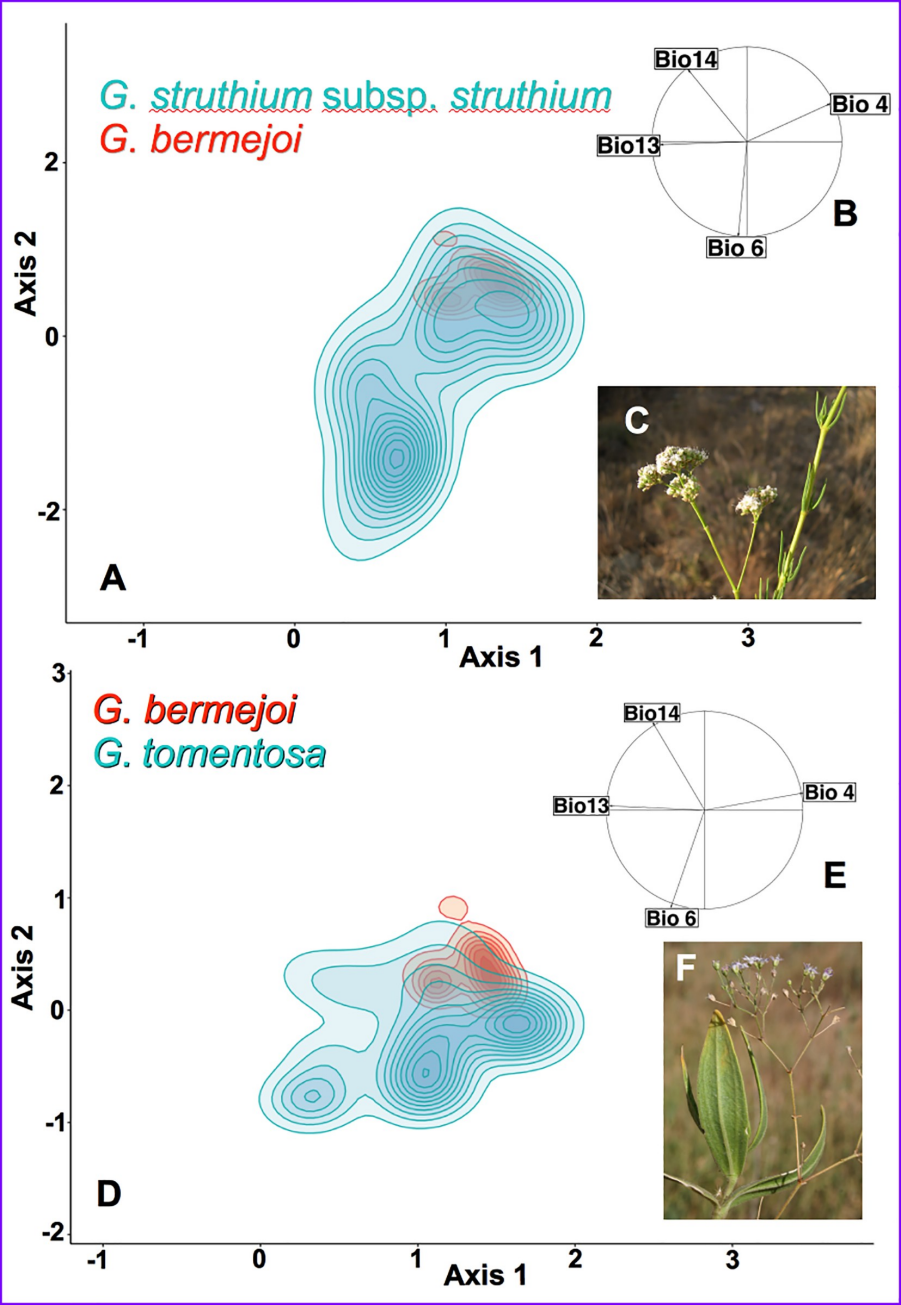
Bioclimatic niches of *G*. *bermejoi* and its parental species (*A* and *D*). The environmental space is defined by the first two axes of the PCA analysis, and it is limited by the minimum and maximum environmental values in the whole study area. The different taxa have been plotted in pairs to make comparisons easier: (*A*) *G*. *bermejoi* (red) vs. *G*. *struthium* subsp. *struthium* (blue); (*D*) *G*. *bermejoi* (red) vs. *G tomentosa*). Note that *G*. *bermejoi* has a narrower niche in both cases. *B* and *E* show the correlation circles for the variables used in the PCAs. The appearance of the parental taxa of *G*. *bermejoi* is shown in *C* (*G*. *struthium* subsp. *struthium*) and *F* (*G*. *tomentosa*).

Both density plots showed remarkable differences in the size of the bioclimatic niche between *G*. *bermejoi* and its two parental species. This can be seen readily when comparing the bioclimatic niches of *G*. *bermejoi* and *G*. *struthium* subsp. *struthium* (Fig 6A), particularly those concerning the area and the centroid projected from the environmental hyperspace. In the case of *G*. *struthium* subsp. *struthium*, this projected area was more than double that for *G*. *bermejoi*. In other words, the set of suitable combinations of bioclimatic variables was much smaller for *G*. *bermejoi* than for *G*. *struthium* subsp. *struthium*. There were also noticeable differences between the bioclimatic niches in the PCA plot of *G*. *bermejoi* and *G*. *tomentosa* (Fig 6B), albeit not as important as those shown for *G*. *struthium* subsp. *struthium*. In this case the projected areas were more closely matched and the centroids were closer, suggesting that the bioclimatic niches for *G*. *bermejoi* and *G*. *tomentosa* were generally more similar.

**Fig 7:**
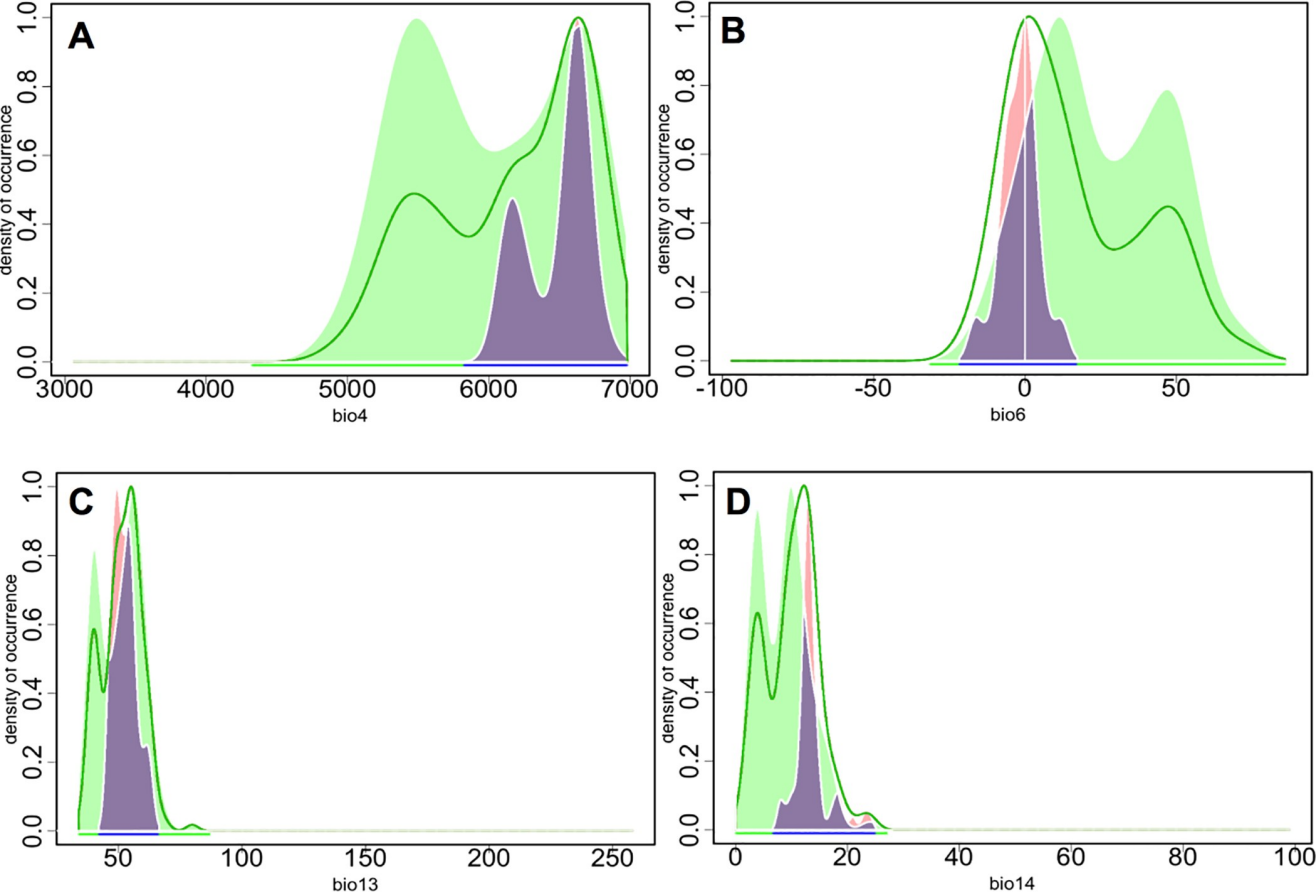
Occurrence density curves for specific bioclimatic variables of *G*. *bermejoi* (pink) and *G*. *struthium* subsp. *struthium* (green). The pale violet areas represent overlapping areas under the curves. Again, the narrow bioclimatic niche of *G*. *bermejoi* becomes evident.

The density curves for specific bioclimatic variables also provide key information concerning the ecological behavior of the species, particularly when two different taxa are plotted in the same graph. Some relevant results were obtained when comparing these curves for *G*. *bermejoi* and *G*. *struthium* subsp. *struthium* (Fig 7), providing data that were relevant to assess the vulnerability of this taxon under a particular scenario of climate change. For instance, the narrower nature of the bioclimatic niche of *G*. *bermejoi* is clear in all cases (Fig 7), which in conjunction with the very steep slopes of the curves for Bio 13 and Bio 14, could suggest high vulnerability for this species. This means that slight changes in the variables can produce dramatic changes in the occurrence density.

In the Mediterranean climate, water stress during the summer is a crucial limiting factor for many plants. Our results suggest that *G*. *bermejoi* has a different ecological behavior to *G*. *struthium* subsp. *struthium*, the latter maintaining a relatively high occurrence density even under conditions of virtually no precipitation during summer. This behavior contrasts with that of *G*. *bermejoi*, where the occurrence density plummeted to 0 below values around 10 mm for the same variable (Bio 14).

The minimum temperature during the coldest month (Bio 6) also deserves some attention due to the extreme temperatures associated with Mediterranean climates. Again, the range of temperatures was much narrower and interestingly, the maximum occurrence densities for *G*. *bermejoi* were around 0 ºC, below that of *G*. *struthium* subsp. *struthium*. We generally found the behavior of *G*. *bermejoi* to be more similar to that of *G*. *tomentosa* (see the occurrence density plots in S1 Fig).

## 4- Discussion

Unusual bedrock and special soil areas, like gypsum or serpentine outcrops, are given high priority for biodiversity conservation. From the point of view of biogeography, such habitats can be considered as edaphic archipelagos **[31]**, and they can be seen as natural laboratories to study evolutionary and ecological processes **[21]**. In addition, they harbor a large number of endemic taxa and rare species. When considering the conservation of these species and communities, it is crucial to assess possible shifts in the optimum climatic areas and the relationship of these changes to their suitable soils. In this study, we examined the evolution of bioclimatically suitable areas for *G*. *bermejoi* under four different scenarios of climate change. As for other species adapted to such specialized soils, if the bioclimatic suitability area does not overlap with the location of suitable soils, this species could be committed to extinction. As such, the bioclimatic niche of this plant was also considered, indicating that this plant has a narrower niche when compared to other closely related taxa, which could make it particularly vulnerable to climate changes. If so, this species should receive special attention in conservation programs. Nevertheless, we should stress that a proper assessment of the extinction risk for this species would require further studies and that this was not the goal of the present study.

The global change that the biosphere will face in the next decades is not limited to climate change. Indeed, fragmentation and reduced habitat quality are other global change drivers **[70]** that will play a very important role in the fate of plant populations and communities in Mediterranean climates. The different climate change scenarios studied here were established according to the four RCP conditions. As there are important differences in the assumptions for these four scenarios, it is not surprising that considerable variation was obtained in the output data for each climate change scenario. We also studied the evolution of bioclimatic suitability values taken at sites were *G*. *bermejoi* is currently found. Under RCP 2.6 conditions, the bioclimatic suitability for *G*. *bermejoi* at its current locations seems to improve noticeably. This might be due to the proposed increase in the minimum temperatures in the coldest month (Bio 6) under this scenario by 2050, remaining at that level until 2070. This could be particularly relevant as the median temperature moves from negative to positive values, probably diminishing the risk of freezing during the winter. The rainfall during the driest month (Bio 14) is also proposed to decrease, yet the median values remain above 10 mm in 2050 (11 mm) increasing again in 2070 (12 mm), which seems to be suitable for this plant to survive under these climatic conditions. According to our models this plant could be capable of dealing with this situation and thus, the models show that at most of the sites where *G*. *bermejoi* is currently found, bioclimatic suitability will be enhanced by 2050 and it will remain stable until 2070.

The outputs under the RCP 4.5 scenario differ with respect to those of RCP 2.6, with somewhat higher predicted Bio 6 values and lower predicted Bio 14 values in 2050 and 2070. The trends in bioclimatic suitability are more or less the same, as again the higher Bio 6 values are more likely to increase suitability by producing more benign winters, whilst the lower Bio 14 values seem to be sufficient to satisfy the plant’s needs. The models define more locations with better bioclimatic conditions around the Ebro Valley, opening the possibility of colonization in those areas.

Under RCP 6.0 there was also a predicted increase in bioclimatic suitability at some of the sites where *G*. *bermejoi* is currently found. However, the medians of suitability appear to be lower than those in the RCP 2.6 and RCP 4.5 scenarios The evolution of Bio 6 and Bio 14 in the RCP 6.0 scenario is clearly less favorable for the plant, with less rainfall and higher temperatures affecting bioclimatic suitability at most of its current locations. In semi-arid regions, water availability is a key factor determining vegetation patterns **[60]**, an abiotic factor that is particularly important during the dry season. Our results show that *G*. *bermejoi* is sensitive to low levels of water during the driest month of the year (Bio 14), in contrast to the behavior of *G*. *struthium* subsp. *struthium*. Indeed, bioclimatic niche studies show that this plant has a narrow range of tolerance for this bioclimatic variable, requiring values above 0, whilst other closely related taxa are capable of enduring summers with no precipitation at all during the driest month. *G*. *struthium* subsp. *struthium* has a bimodal density distribution with one of its maxima located at summer precipitation (Bio 14) values beyond the range of *G*. *bermejoi* tolerance. In other words, *G*. *struthium* subsp. *struthium* seems to feel at home during dry summers that *G*. *bermejoi* would not endure. The flowering period for *G*. *bermejoi* spans from June to September, a period that includes the driest month in the Mediterranean climate of the Iberian Peninsula **[71]**. Water stress could seriously affect seed formation and in terms of germination, the opportunistic strategy adopted by this plant **[48]** could be affected by periods of severe drought. The evolution of bioclimatic suitability under the RCP 8.5 scenario and the response curves generated by MaxEnt suggest the presence of an eco-physiological threshold of *G*. *bermejoi* around 10 mm of rainfall for Bio 14. Precipitation levels below this value will most likely seriously affect the bioclimatic suitability of this species.

We also studied the evolution of bioclimatic suitability in all the gypsum outcrops in the Iberian Peninsula. At some of these locations where *G*. *bermejoi* is not currently present, its suitability was enhanced under the different RCP scenarios. It is important to note that this does not mean that *G*. *bermejoi* could reach those areas in the period of time contemplated, although historically there is strong evidence for migration and episodes of colonization for this and other related *Gypsophila* taxa **[23, 72]**. Indeed, it is difficult to establish whether or not these areas could be colonized by this plant within this time span (2070). The key question here is whether the adaptation or redistribution of *G*. *bermejoi* can run at the same pace as the changes in the areas of bioclimatic suitability. If the answer to that question is no, the preservation of this species under climate change may perhaps require conservation strategies, such as managed relocation **[22]**.

Habitat quality and fragmentation are also relevant factors when it comes to extinction risk. In gypsum soils, there are some insightful results concerning habitat quality. This factor, as measured by plant cover and soil nutrient content, affects the responses of *Centaurea hyssopifolia* to reduced precipitation **[22]**, a gypsophile species that like *G*. *bermejoi* is also endemic to the Iberian Peninsula. Habitat fragmentation is another considerable threat for the conservation of genetic diversity in many plant populations **[22]**. Some studies assessing the effects of both fragment size and connectivity in gypsum soils indicate that habitat connectivity is crucial in the fragmentation process **[73]**. Special soil endemics live in naturally-fragmented habitats, which may lead to genetic erosion and higher vulnerability to fragmentation. **[74]**. Yet again, this is difficult to demonstrate, because their evolution in a fragmented landscape could increase their resilience to the effects of further fragmentation **[13]**. Nevertheless, there is evidence of the effects of connectivity on the fitness of gypsophile species, showing the importance of including habitat connectivity in management and conservation strategies for *G*. *bermejoi*, and other species and communities associated with specialist soils **[75]**.

As indicated above, trying to foresee the fate of this taxon is a challenging task, its survival being linked to the concurrence of bioclimatic and soil suitability. Soil specialization is often associated with the acquisition of traits related to the tolerance of drought and nutrient imbalances. This circumstance would confer them additional advantages to confront climate change. Indeed, our results appear to indicate that in most of the climatic change scenarios, *G*. *bermejoi* will probably behave more like a hardy survivor than an early victim **[22]**.

## 5- Conclusions

From the results obtained, we can draw the following conclusions:

- The output of the four RCPs differ widely and as a result, we have a wide range of potential answers to the questions we set out to address. In most of the climatic change scenarios *G*. *bermejoi* seems to behave like a hardy survivor more than an early victim. The most optimistic scenario (RCP2.6) is a more or less stable scenario where the potential bioclimatic area still overlaps with the suitable soils in 2050 and 2070. Bioclimatic suitability increases at most of the locations where *G*. *bermejoi* is currently found. The most pessimistic scenario (RCP8.5) is that which changes most and while suitability would be predicted to improve by 2050. It then appears to plummet markedly by 2070. Some studies suggest RCP 8.5 is a likely scenario **[76]** and if this were the case, conservation measures would be needed to preserve this species, perhaps including managed relocation.
- Even under the worst climate change scenario, the approach adopted enables us to locate future bioclimatic refuges for *G*. *bermejoi*. This information should be taken into account when designing future conservation plans.
- In several RCP scenarios there are gypsum outcrops where *G*. *bermejoi* is absent but where bioclimatic suitability will be enhanced. Regardless of the evidence of past migration and colonization events for this and closely related taxa, we cannot assess whether this plant will be able to reach these future suitable bioclimatic areas. More information is required concerning seed dispersal dynamics for this taxon and gypsum outcrop connectivity.
- When the trends for bioclimatic suitability under different RCPs were compared and density curves were generated, there appears to be a physiological threshold of around 10 mm of rainfall in the driest month (Bio 14). Again, further studies will be necessary to shed more light on this issue.
- Habitat connectivity is a crucial issue regarding management and conservation strategies, not only for *G*. *bermejoi* but also for other soil specialist species and communities.

## Supporting information

S1 Fig Occurrence density curves for specific bioclimatic variables of *G*. *bermejoi* (pink) and *G*. *tomentosa* (green). The pale violet areas represent overlapping areas under the curves. We generally found the behavior of *G*. *bermejoi* to be more similar to that of *G*. *tomentosa*.
(TIF)
