# Supplementary figures and images for: Four climate change scenarios for Gypsophila bermejoi G. López (Caryophyllaceae) to address whether bioclimatic and soil suitability will overlap in the future
Source: PLoS One. 2019 Jun 13;14(6):e0218160. doi: 10.1371/journal.pone.0218160 (PMC6564006; doi:10.1371/journal.pone.0218160)

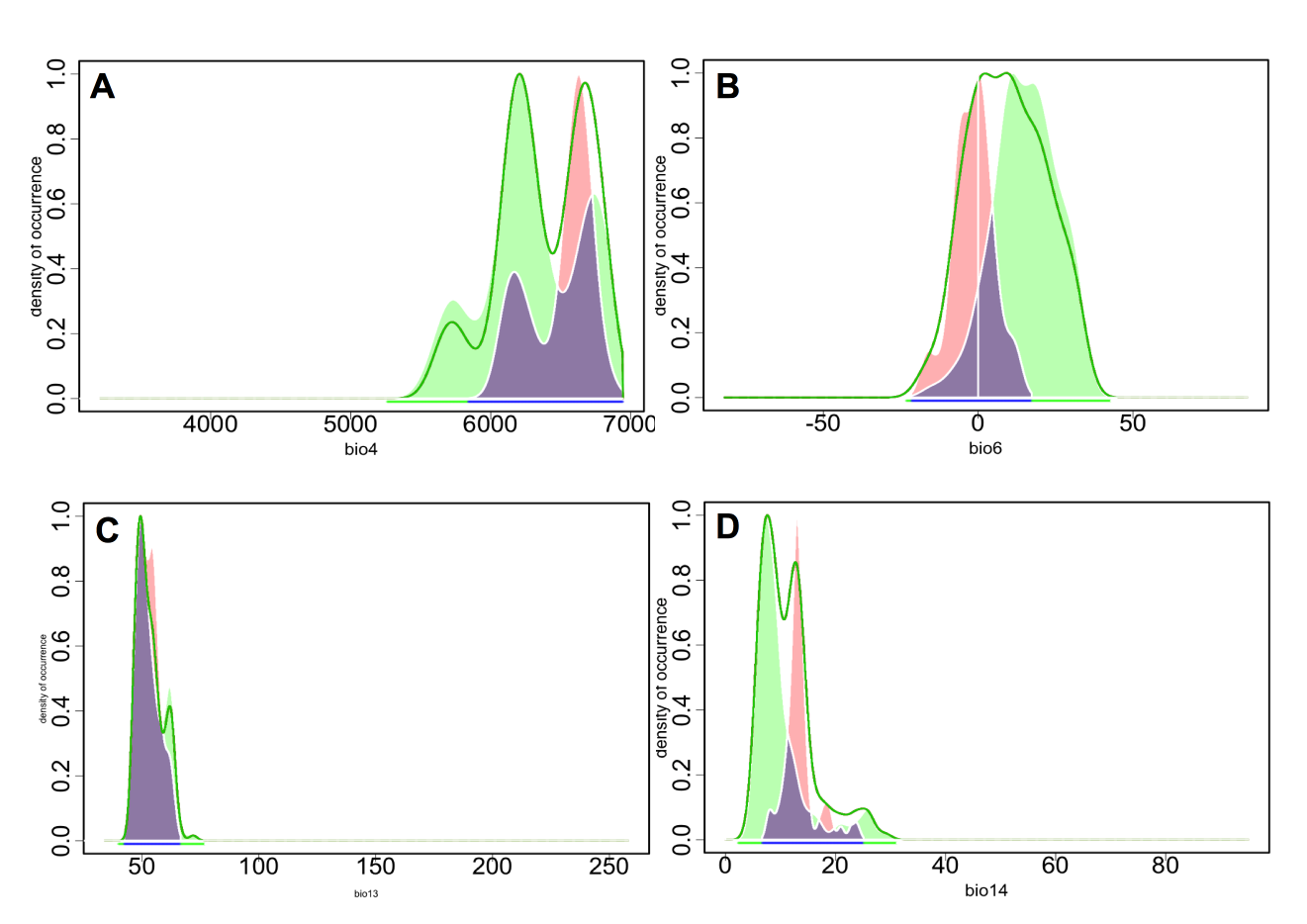

Supplement: S1 Fig — The pale violet areas represent overlapping areas under the curves. We generally found the behavior of G. bermejoi to be more similar to that of G. tomentosa. (TIF) [file pone.0218160.s001.tif]
